# The effects of opioid tapering on select endocrine measures in men and women with head and neck cancer—a longitudinal 12-month study

**DOI:** 10.1097/PR9.0000000000001183

**Published:** 2024-09-13

**Authors:** Thomas F. Kallman, Emmanuel Bäckryd

**Affiliations:** Pain and Rehabilitation Center, and Department of Health, Medicine and Caring Sciences, Linköping University, Linköping, Sweden

**Keywords:** Opioid tapering, Opioid-induced hypogonadism, Endocrinopathy, Gonadal hormones, Testosterone, Cancer-related pain

## Abstract

**Introduction:**

Opioid treatment may affect endocrine measures in humans either through centrally or peripherally mediated mechanisms. There is a general lack of longitudinal studies examining endocrine measures in opioid-treated patients.

**Objectives:**

To longitudinally follow the levels of select endocrine measures in men and women with head and neck cancer for 1 year, who after having completed radiotherapy began tapering opioids.

**Methods:**

This was a prospective, longitudinal, observational study. Testosterone and estradiol were measured in men and women, respectively. Follicle-stimulating hormone (FSH), luteinizing hormone (LH), dehydroepiandrosterone sulfate (DHEAS), and prolactin were measured in both sexes. Women were grouped based on if premenopausal or postmenopausal. Samples were collected when opioid tapering started and at 1, 3, 6, and 12 months after tapering start. Daily opioid doses at the same time points were registered.

**Results:**

Twenty-five men and 12 women were followed for 12 months. In men, testosterone levels increased significantly during the first month after opioid tapering started (*P* < 0.001). Levels of testosterone, FSH, DHEAS, and prolactin changed significantly in men during the study period. A moderate correlation between opioid dose reduction and testosterone level increase in men aged ≤60 years was found (*r*_s_ = −0.577, 95% CI −0.854 to −0.044, *P* = 0.039). In postmenopausal women (n = 10), levels of FSH and LH changed significantly during the study period.

**Conclusion:**

Previously known effects of opioids on endocrine measures in humans seem to be reversible as select endocrine measures changed significantly in men and postmenopausal women after opioid tapering was initiated.

## Abstract 2

Supplemental Digital Content is Available in the Text.

Previously known effects of opioids on select endocrine measures seemed to be reversible after opioid tapering started. Endocrine measures changed significantly during the study period.

## 1. Introduction

Cancer-related pain in patients with advanced cancer and those undergoing anticancer treatment is common; one-third of cancer patients estimate their pain to be moderate or severe.^41^ When comparing different cancer types, patients with head and neck cancer (HNC) have reported the highest pain prevalence, ie, 70%.^41^ Head and neck cancer treatment is multimodal, often requiring surgery, chemotherapy, and radiotherapy (RT).^6,25^ Radiotherapy may induce oral mucositis, leading to pain and affecting quality of life negatively.^25,27^ Subsequently, opioids are central for the management of cancer-related pain and improvement of quality of life in general,^13^ and for HNC patients in particular, systemic opioids are “almost always necessary for pain relief.”^24,27^ Many HNC survivors report pain after treatment and strong opioids have been found to be used by a minority of these patients for many years after the cancer diagnosis.^31^

Opioid treatment suppresses the levels of most hormones, except for prolactin that shows increased levels.^1–3,7,10,14,42^ Alterations may be of both acute and chronic character.^3,20,42^ Despite this knowledge, opioid-induced endocrine complications may often be overlooked.^3^ Opioid-induced endocrine alterations can be centrally or peripherally mediated, ie, effectuated through impact on the hypothalamic–pituitary axes or through direct effect on endocrine glands, respectively.^4,11,20,42^ More specifically, the central mechanism is believed to be that µ-opioid receptors in the hypothalamus interfere with the pulsatile secretion of hormones, and the peripheral mechanism through specific opioid receptors in the endocrine organs.^4,11,22^ Route of opioid administration as well as opioid dose may also be important factors to account for.^9,12,29,30,33^

There is a general lack of longitudinal studies on the subject of opioid tapering and endocrine measures. This prompted McWilliams et al.^26^ to, already in 2014, conclude that “a longitudinal study examining the impact of opioids on the hypogonadal axis would be of interest.” A more recent review on opioid treatment and endocrine measures in patients with cancer-related pain found 5 studies to include for analysis, none of which were of longitudinal design.^1^

To the best of our knowledge, no previous study has longitudinally investigated the effect of opioid tapering on endocrine measures in patients with cancer. Thus, the aim of the present study was to longitudinally follow the levels of select endocrine measures in men and women with HNC who, after having completed RT and RT-induced oral mucositis was subsiding, began tapering off opioid treatment.

## 2. Methods

### 2.1. Clinical setting and study population

The Pain and Rehabilitation Clinic (PRC) at Linköping University Hospital has a well-established and formalized clinical cooperation with the Radiotherapy Clinic at the same hospital. Patients with HNC eligible for adjuvant RT receive RT daily for 6 to 8 weeks and often develop painful oral mucositis as a treatment side effect.^25,27^

During 2016–2021, patients with HNC who completed RT and began tapering opioids when oral mucositis began to subside were recruited for study inclusion. Exclusion criteria were (1) rapid metastasizing disease and short expected survival time and (2) inability to give informed consent. Female participants were asked about menstruation status and registered as either premenopausal or postmenopausal.

### 2.2. Pain management strategy and opioid data

The clinical routine for pain management conducted by the PRC for this patient group has been described in detail in previous studies.^25,34–36^ In summary, specialized pain nurses from the PRC assessed the patients regularly with the support of pain specialists. A numeric rating scale (NRS) from 0 (no pain) to 10 (most intense pain imaginable) during the previous 24 hours was used clinically to guide pharmacologic treatment. If the pain originated from the oral cavity or pharynx, it was assessed to be nociceptive pain type, and if patients scored pain intensity NRS >6, a strong long-acting opioid was prescribed. In the present study, all patients fulfilling these criteria were prescribed the lowest dose of fentanyl patch available. If breakthrough pain occurred, short-acting opioids such as oxycodone or morphine were later added.

Pain nurses registered opioid doses at each time point, as described below. Similar to previous studies conducted by the present authors,^18,36^ we chose to convert opioid doses to oral morphine equivalents (OME) in milligrams (mg) per day in accordance with the equivalence table found in the European Pain Federation position paper on appropriate opioid use by O'Brien et al.^28^ Opioid tapering was conducted according to the PRC's clinical routines. In summary, this entails that opioid tapering is initiated 2 to 3 weeks after radiation treatment is completed. The rate at which tapering is conducted is dependent on clinical factors, eg, pain interfering with sleep and/or the ability to eat or drink. For most study subjects, the fentanyl patch was tapered first, followed by short-acting opioids, if applicable.

### 2.3. Blood sample collection and reference intervals

Total testosterone and estradiol were analyzed for men and women, respectively. Follicle-stimulating hormone (FSH), luteinizing hormone (LH), prolactin, and dehydroepiandrosterone sulfate (DHEAS) were analyzed in both men and women.

Blood samples were collected as regular clinical blood samples, ie, they were collected by health care staff either at the patient's primary health care center or at Linköping University Hospital's testing center and not by research staff. Consequently, the hours during which patients could have blood drawn were regular office hours.

Blood samples were collected:

(1) When patients started tapering opioids (T0)
(2) One month after T0 (T1)
(3) Three months after T0 (T3)
(4) Six months after T0 (T6)
(5) Twelve months after T0 (T12)

Samples were analyzed by the clinical chemistry department at Linköping University Hospital using immunoassays and electrochemiluminescence (ECL) with Roche Cobas E602 Immunology Analyzer (Basel, Switzerland). Dehydroepiandrosterone sulfate was sent to Karolinska University Laboratory in Stockholm and analyzed with immunoassays and ECL. Reference intervals used to determine if blood samples were within normal ranges or pathologic for men and women are available in Supplementary Information, Table S1 and Table S2, http://links.lww.com/PR9/A242, respectively.

### 2.4. Statistics

The IBM Statistical Package for the Social Sciences (SPSS, IBM Corporation, Somers, NY) version 29.0 was used. Data are reported as median (25th–75th percentile), unless otherwise specified. The paired samples sign test was used for 2 related median comparisons. Friedman test used for multiple related samples. McNemar test was used for 2 paired categorical samples. Cochran Q test was used for multiple related categorical samples. Spearman rank correlation test (*r*_s_) was used for correlation analysis, and confidence intervals for Spearman test were estimated in SPSS using the method proposed by Caruso and Cliff.^5^ *P* ≤ 0.05 was considered statistically significant for all tests.

Missing values were handled by use of multiple imputation (MI).^17,19^ In accordance with the ethical authorization, all patients who completed at least 6 months of participation were included in the final analysis. Men and postmenopausal women were missing 5% and 9% of variables during the study period, respectively. Consequently, in the imputation models, we chose a level of 5 imputations and 9 imputations for men and postmenopausal women, respectively.^17^ Age, weight, days on opioid treatment at T0, radiation amount (Gray) at T0, adjuvant chemotherapy (yes/no), as well as OME, endocrine measures, and time of day for blood test during T0–T12 were used as variables in the MI models. We used SPSS’ automatic function for selection of imputation method, which subsequently in all models were linear regression.

Because of many variables not showing a normal distribution, we chose to present data as median (25th–75th percentile). As a result of this, depending on statistical test used, SPSS does not compute a pooled median value. Subsequently, for all data and analyses, we present the first imputation's median and *P*-value for all results.

For all available patient data from men and postmenopausal women (n = 28 and n = 13, respectively) in T0 and T1, we calculated delta (Δ) values for OME and endocrine measures by calculating T1 values subtracted by T0 values. Because of an increased number of subjects available for analysis in T1 and subsequently also increased number of missing values (10% and 17% for men and postmenopausal men, respectively), separate MI models were performed for any missing values in the delta calculations, with 10 and 17 imputations performed for men and postmenopausal women, respectively. The same variables and method mentioned above for the first MI model were used, and for each respective MI, the first imputation's median and *P*-value were reported.

### 2.5. Ethics

Ethical approval was granted by the Regional Ethics Committee in Linköping, Sweden (Dnr 2015/428–31). All patients gave informed consent to participate in the study before study inclusion. No study protocol was published before study initiation.

## 3. Results

**Figure 1.**
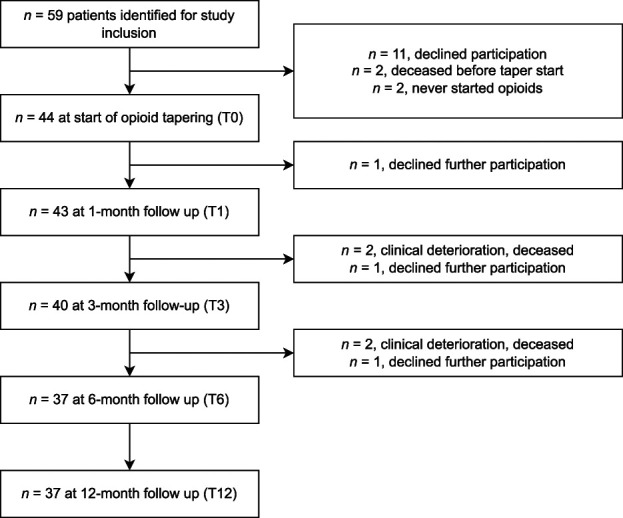
Flow chart of study population during the study period with reasons for study subject discontinuation. Thirty-seven patients were followed for the entire study period, ie, until 12-month follow-up (T12).

A total of 44 patients were included in the study and were hence assessed at T0, and 37 of them (ie, 84%) were followed for 1 year (ie, up to T12) (Fig. 1). Demographic and basic clinical characteristics, including opioid doses in OME, in the 28 male and 16 female participants who were assessed at T0 are shown in Table 1. Of these, 25 male and 12 female participants had endocrine measures that were followed for 1 year.

**Table 1:** Demographic data and basic clinical characteristics of the 28 men and 16 women at start of opioid tapering (T0) included in the study.

Variable	Men (n = 28)	Women (n = 16)
Age (y)	61 (55–66)	59 (49–68)
Weight (kg)	84 (77–99)	71 (56–78)
Days on opioids before taper start	56 (47–94)	72 (54–103)
Radiotherapy, total amount Gray	68 (68–68)	68 (68–68)
Cancer in epipharynx, n (%)	1 (4%)	0 (0%)
Cancer in oropharynx, n (%)	14 (50%)	8 (50%)
Cancer in hypopharynx, n (%)	13 (46%)	8 (50%)
Adjuvant chemotherapy, n (%)	7 (25%)	3 (19%)

Data are presented as median (25th–75th percentile) or number (percentage).

### 3.1. Endocrine measures in men

Between T0 and T1, median OME decreased by 67% from 210 (105–305) mg per day to 70 (0–110) mg per day, and in parallel, median testosterone increased by 47% from 9.5 (4.2–14.0) nmol/L to 14.0 (11.0–18.8) nmol/L during the same period (*P* < 0.001) (Table 2). The percentage of men on opioid treatment at each time point decreased from 100–72–12–12–8% between T0 and T12. In parallel, levels of testosterone, FSH, DHEAS, and prolactin (PRL) changed significantly during the study period, whereas levels of LH did not change (Table 2).

**Table 2:** Oral morphine equivalents, endocrine measures, and time when samples were drawn in men followed for 1 year (n = 25).

Variable	T0	T1	T3	T6	T12	*P*
OME (mg)	210 (105–305)	70 (0–110)	0 (0–0)	0 (0–0)	0 (0–0)	<0.001*†
Testosterone (nmol/L)	9.5 (4.2–14.0)	14.0 (11.0–18.8)	14.0 (9.9–16.5)	14.0 (10.5–17.0)	13.0 (9.8–15.5)	<0.001*†
FSH (IU/L)	6.4 (4.8–12.5)	9.8 (6.2–15.5)	8.2 (5.1–15.0)	8.9 (5.7–11.9)	5.6 (4.2–7.4)	<0.001*
LH (IU/L)	5.6 (4.1–7.3)	5.7 (4.4–7.3)	5.7 (3.8–7.7)	5.3 (4.0–6.6)	5.1 (3.5–6.5)	0.287
DHEAS (µmol/L)	1.3 (0.8–2.4)	2.0 (1.1–3.1)	2.2 (1.4–3.1)	1.9 (1.5–2.6)	2.0 (1.5–3.1)	0.004*
PRL (mIU/L)	340 (240–560)	220 (175–415)	220 (185–280)	270 (145–360)	260 (175–296)	0.019*
Time of day test was drawn (24 h)	9 (9–10:30)	9 (9–10)	9 (8–10)	9 (8:45–10)	9 (8:45–10)	0.525

Data are presented as median (25th–75th percentile).

* Statistical significance at the 0.05 level.

† All imputations have the same *P*-value.

DHEAS, dehydroepiandrosterone sulfate; FSH, follicle-stimulating hormone; LH, luteinizing hormone; OME, oral morphine equivalents; PRL, prolactin.

At T0, 36% of men had testosterone levels below the lower limit of the reference interval, all of which had normalized at T1 (*P* = 0.004). Apart from T0 and one male who at T3 showed a testosterone level above the upper reference interval, testosterone levels remained within the reference intervals for all men during the remainder of the study period (*P* < 0.001). Concerning FSH, 20% to 32% of men had values above the upper limit of the reference interval between T0 and T6, all of which subsequently had normalized at T12, except one (*P* = 0.02). For LH, 24% of men had values outside of the reference interval at T0. Of these, 4 values were above and 2 values were below the reference interval. At T12, 8% of men had LH values above the upper reference interval (*P* = 0.406). Thirty-six percent of men had DHEAS levels below the lower limit of the reference interval, a proportion which decreased during the study period to 20% at T12 (*P* = 0.039). For PRL, 36% of men had levels above the upper reference interval at T0, a proportion that decreased during the study period to 12% (*P* = 0.007).

All in all, testosterone and DHEAS levels increased quickly during the first month of opioid tapering and remained relatively constant thereafter, as reported in Table 2. Prolactin levels also changed relatively quickly (ie, decreased). Luteinizing hormone did not change. The pattern for FSH was more difficult to interpret.

### 3.2. Endocrine measures in postmenopausal women

Ten women reported that they were postmenopausal, which was confirmed by unmeasurable levels of estradiol (ie, <44 pmol/L) in all 10 women during the entire study period. Between T0 and T1, median OME decreased by 78% from 130 (108–198) mg per day to 28 (0–73) mg per day. Seven women remained on opioid treatment at T1, which decreased to 1 woman at T3 (*P* = 0.031). One woman remained on opioid treatment during the remainder of the study period; however, she decreased her OME from 350 mg per day at T0 to 80 mg per day at T12, ie, a 77% reduction in OME during the study period. Significant changes for levels of FSH and LH were found, which increased during the study period (Table 3). There was also a significant change in time of day when postmenopausal women had tests drawn during the study period (Table 3). The proportion of endocrine values outside the lower or upper level of the reference intervals varied between 10% and 20% for FSH, LH, and DHEAS during the study period, with no significant changes in proportion of tests above or below the reference intervals. Six women had levels of PRL above the reference interval at T0, and all postmenopausal women had normalized levels of PRL by T12 (*P* = 0.012).

**Table 3:** Oral morphine equivalents, endocrine measures, and time when samples were drawn in postmenopausal women followed for 1 year (n = 10).

Variable	T0	T1	T3	T6	T12	*P*
OME (mg)	130 (108–198)	28 (0–73)	0 (0–0)	0 (0–0)	0 (0–4)	<0.001*†
Estradiol (pmol/L)	<44 (44–44)	<44 (44–44)	<44 (44–44)	<44 (44–44)	<44 (44–44)	n/a
FSH (IU/L)	52 (31–105)	76 (63–85)	80 (70–99)	89 (65–105)	82 (73–93)	0.03*
LH (IU/L)	23 (7.7–43)	35 (33–42)	45 (31–58)	42 (30–65)	40 (34–51)	0.019*
DHEAS (µmol/L)	2.4 (1.0–3.8)	3.4 (1.6–4.8)	3.0 (1.7–4.0)	3.3 (1.7–4.4)	3.5 (2.2–4.8)	0.168
PRL (mIU/L)	730 (198–2342)	230 (173–320)	300 (200–628)	270 (203–485)	266 (220–324)	0.086
Time of day test was drawn (24 h)	10:30 (9–12)	10 (9–11)	11:30 (10:30–14:15)	9:15 (8.0–11:15)	11 (9:15–11:30)	0.037*

Data are presented as median (25th–75th percentile).

* Statistical significance at the 0.05 level.

† All imputations have the same *P*-value.

DHEAS, dehydroepiandrosterone sulfate; FSH, follicle-stimulating hormone; LH, luteinizing hormone; OME, oral morphine equivalents; PRL, prolactin.

### 3.3. Endocrine measures and oral morphine equivalents in premenopausal women

Two women who reported that they were premenopausal were followed for the entire study period, and these 2 were also the youngest female study participants (<50 years of age). Because of the low number of premenopausal women available for analysis, we have chosen to highlight the changes in endocrine measures for these 2 women as case reports, see Supplementary Information, Figure S1 and Figure S2, http://links.lww.com/PR9/A242.

### 3.4. Correlations between endocrine measure and oral morphine equivalent delta values in men and postmenopausal women

**Figure 2.**
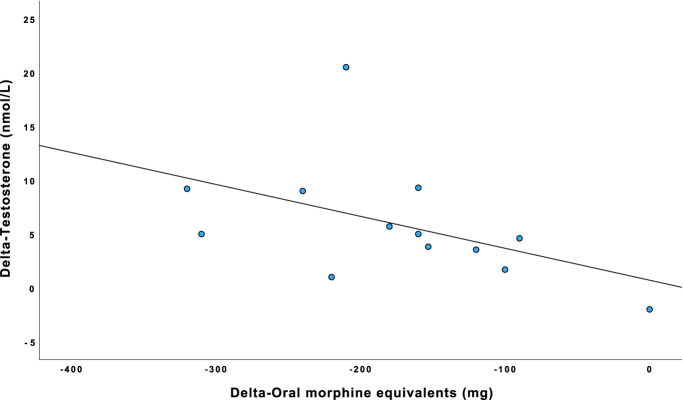
Scatter plot of the relationship between delta (Δ)-testosterone and Δ-oral morphine equivalents (OME) in mg per day in men aged <60 years (n = 12). Δ-values were calculated by subtracting all available values at tapering start (T0) from values at 1-month follow-up (T1). All subjects lowered their opioid doses between T0 and T1, except one who remained on the same dose at T1. Please note that the *x*-axis Δ-OME is negative, ie, a negative value stands for a reduction in opioid dose between T0 and T1.

For all men with complete endocrine and OME data at T1 (n = 28), we found no statistically significant correlation between OME and endocrine delta(Δ)-values. When stratifying for age, we found a significant correlation between Δ-OME and Δ-testosterone, *r*_s_ = −0.577 (95% CI −0.854, −0.044, *P* = 0.039) in men aged ≤60 years (n = 13) (Fig. 2). In men aged >60 years (n = 15), the correlation between Δ-OME and Δ-testosterone was *r*_s_ = −0.054 (95% CI −0.536, 0.456, *P* = 0.849).

For all postmenopausal women with complete endocrine and OME data at T1 (n = 13), we found no significant correlations between OME and endocrine measures.

## 4. Discussion

This small yet *novel* longitudinal study illustrates the potential effects that opioid tapering may have on levels of select endocrine measures in men and postmenopausal women. A minor portion of variables were missing values, and thus imputed by multiple imputation, a statistically accurate method even for small sample sizes.^17,19^ In men, we found that during the first month of opioid tapering, a decrease in OME by two-thirds was paralleled by a quick increase in levels of testosterone. In addition, 36% of men had testosterone levels below the lower clinical reference interval, levels which all normalized by 1-month follow-up. For men aged ≤60 years, we found a moderate correlation between increase in testosterone levels and decrease in OME. In postmenopausal women, only levels of FSH and LH changed significantly over time, but there was also a significant change in time of day when blood tests were drawn. We also found that DHEAS levels quickly increased in men, and in postmenopausal women, there was a similar nonsignificant trend in DHEAS levels. For PRL, we found that levels in men significantly decreased, and postmenopausal women showed a similar nonsignificant trend. It is possible that the lack of significant findings in postmenopausal women is because of lack of statistical power.

Thirty-six percent of men had low blood testosterone levels at opioid tapering start, indicating endocrinological signs of opioid-induced hypogonadism, a prevalence that falls within the wide prevalence span found in previous studies.^2,8,15^ Testosterone levels increased significantly within the first month after opioid tapering was initiated and had normalized in all men by T1. Taken together with the fact that almost three-fourths of men still used opioids at T1, our results indicate that even if opioids are not tapered completely, opioid-induced changes in testosterone levels seemed to reverse quickly when opioid doses were decreased T0 to T1.

Fentanyl patches have previously been reported to have the highest odds of inducing androgen deficiency.^33^ Our patients used predominantly fentanyl patches,^36^ which thus may have contributed to the levels of testosterone we report. In addition, opioid dose should also be accounted for because previous studies have reported dose-related interactions, ie, as opioid doses increase, testosterone levels decrease.^1,9,12^ Although the opioid doses in both men and postmenopausal women at tapering start may at first sight be viewed as high, it is apt to remember that our study population consists of patients with cancer-related pain. As such, we view the median doses we report at tapering start as both expected and acceptable from a clinical viewpoint. Future longitudinal studies would do well to include and compare different routes of opioid administration and how this may possibly affect both suppression and reversal of suppressed testosterone levels.

We found a moderate correlation between reduction in OME and increase in testosterone levels in men aged ≤60 years. Conversely, we found no significant correlation between these factors for men aged >60 years. Age seems to be an important factor to account for, as our results indicate that men aged ≤60 years may be more susceptible to the suppressing effects opioids may have on levels of testosterone. Furthermore, as indicated by Figure 2 and in similarity to that found by Eshraghi et al.,^12^ we view that this correlation further strengthens the plausibility that there exists a possible linear dose–response relationship between reduction in opioid dose and in increase in testosterone level, and our results may offer an initial insight into the magnitude of this relationship.

Given prior knowledge of the effects of opioids on levels of LH and FSH,^8,14,42^ taken together with that low FSH levels have previously been reported in cancer patients,^1^ we expected to see low levels of LH and FSH at tapering start. Congruently, we found that FSH levels increased significantly after opioid tapering was initiated. Interestingly, however, levels of LH did not change and were stable for the entirety of the study period. This was somewhat surprising because one would expect LH to increase when the opioid-induced hypothalamic-pituitary-gonadal inhibition is decreased.^4,8,22^ It is, however, worth to remind oneself that opioids have been suggested to have a direct effect on the gonads.^4,20^

For postmenopausal women, estradiol levels did not change and remained unmeasurable for the whole study period regardless of opioid dose, confirming that these women were postmenopausal. We found that levels of FSH and LH were lower at opioid tapering start than at study conclusion, congruent with previous reports of low LH levels in opioid-treated postmenopausal women.^39^ Our results may indicate that the inhibitory effects of opioids on levels of both FSH and LH in postmenopausal women were reversible when opioid dose was decreased. Because of the small sample size of women and possible impact of when during the day blood tests were drawn, our results should be interpreted carefully.

Circulating dehydroepiandrosterone and it's sulfate ester, DHEAS, are produced by the adrenal cortex^40^ and are precursors of testosterone and oestrogen.^1^ As such, they are an important source of sex steroids in men and women.^23^ In 2006, Daniell^10^ found that opioid-treated patients had lower levels of DHEAS compared with control subjects in a dose-related pattern. We found that 36% of men had DHEAS levels below the lower reference interval at taper start, with significant changes in this proportion during the study period. Levels of DHEAS in men also increased significantly during the study period. For postmenopausal women, a similar nonsignificant trend in DHEAS levels was noted. Taken together, our results indicated that opioid-induced suppression of DHEAS, similar to that found by Daniell,^10^ seem to be reversible after opioid tapering is initiated. Unlike Daniell, however, we were not able to find any correlation between OME and DHEAS levels. Furthermore, unlike other adrenal hormones, DHEAS has shown small circadian variation,^37^ which may mitigate any impact that time of day our patients had blood tests drawn, particularly applicable for DHEAS levels we found in postmenopausal women.

Acute opioid administration increases PRL levels,^42^ both in men and in postmenopausal women.^3^ Opioid-induced increases in pituitary PRL secretion may exacerbate testosterone suppression.^2^ We found that PRL levels decreased significantly in men, and in postmenopausal women, there was a nonsignificant trend toward lower PRL levels after opioid tapering started. Given these results, we consider that they reflect that opioid-induced hyperprolactinemia was reversible after opioid tapering started.

An obvious limitation in the present study is the small sample size. It is possible that there were significant changes in endocrine measures in our patients that we have not been able to identify. The small sample size was, in part, because of the organizational impact of the SARS-2-CoV pandemic on health care organization, priorities, and ongoing research.^38^ As such, our results in both men and women should be interpreted with care. Blood sample collection was not standardized as to time of day for collection, which could have affected endocrine levels we found. This limitation may be of less significance for men as Table 2 shows that patients consistently had blood samples drawn at the same time. For postmenopausal women, however, there was a significant change in time when blood tests were drawn during the study period. Consequently, we deem it possible that this fact may have affected levels of endocrine measures in postmenopausal women. The underlying cancer or cancer treatment may have altered the endocrine measures we found.^16^ Changes in endocrine measures may have coincided with an improvement in patients' general overall condition, which may have affected the changes in endocrine measures we found. Radiotherapy-induced mucositis may lead to weight loss in HNC patients,^32^ and weight loss may increase testosterone levels.^21^ Apart from weight data at inclusion, weight was not registered T1 to T12, and we therefore cannot rule out that this aspect may be present in our results. We did not adjust for common hypogonadism comorbidities, such as obesity, hypertension, hyperlipidemia, and diabetes.^8,33^ Taken together, the above may have affected the internal validity of our study. Finally, because of the small study population and well-defined patient group being studied, there are questions regarding generalizability of our results to other patient groups. Despite these limitations, we view that our results offer valuable initial insights into an area of research where there is a paucity of data.

In conclusion, this longitudinal study found that levels of testosterone, FSH, DHEAS, and PRL changed significantly in men during the study period after opioid tapering was started. Levels of FSH and LH in postmenopausal women also changed significantly during the study period after opioid tapering was started. Our results indicate that previously known effects of opioids on select endocrine measures in humans seem to be reversible after opioid tapering is initiated. The 2 premenopausal case reports presented in Supplementary Information, http://links.lww.com/PR9/A242, are also consistent with this view. Future longitudinal studies would do well to include larger sample sizes of both men and women with a more even distribution between premenopausal and postmenopausal women, to evaluate hypogonadism symptoms, to account for potential underlying endocrine conditions and common hypogonadism comorbidities, and to examine the subject matter at hand in chronic *noncancer* pain populations.

## Disclosures

The authors have no conflict of interest to declare.

## Appendix A. Supplemental digital content

Supplemental digital content associated with this article can be found online at http://links.lww.com/PR9/A242.
