# Supplementary material for: The effects of opioid tapering on select endocrine measures in men and women with head and neck cancer—a longitudinal 12-month study
Source: Pain Rep. 2024 Sep 13;9(5):e1183. doi: 10.1097/PR9.0000000000001183 (PMC11404959; doi:10.1097/PR9.0000000000001183)
Supplement: Supplementary file 1 [file painreports-9-e1183-s001.pdf]

**Supplementary Information** to "*The effects of opioid tapering on endocrine measures in men and women with head and neck cancer – a longitudinal 12-month study*"

**Authors**: Thomas F Kallman[1], MD and Emmanuel Bäckryd[1], MD, Associate Professor

1) Pain and Rehabilitation Center, and Department of Health, Medicine and Caring Sciences, Linköping University, Linköping, Sweden.

**Number of pages in supplement**: 9

**Number of figures in supplement**: 2

**Number of tables in supplement**: 2

## Methods S1

Blood sample reference intervals used in to determine if blood samples were to be considered normal or pathologic are available in **Table S1** and **Table S2**.

Due to the low number of premenopausal women included in the present study ($n$ = 2) and consequent lack of statistical power, inferential statistics for these two women were not performed. Instead, we chose to present them below as case reports with figures detailing changes in endocrine measures during the study period.

## Results S1

Both case reports were below 50 years of age, both received radiotherapy aimed at the neck/throat/hypopharynx totaling 68 Gray, and neither received adjuvant chemotherapy.

*Case report #1.*

This patient used opioids for 52 days before opioid tapering began. Oral morphine equivalents (OME) decreased 18% between taper start (i.e., T0) and one month follow up (i.e., T1). Between T1 and three-month follow-up (i.e., T3), OME decreased an additional 78%. In parallel, during the same time periods, estradiol increased by 45% and 547%, respectively. For an overview of how endocrine measures changed over time during the study period, see **Figure S1**. Blood samples of prolactin (PRL) and dehydroepiandrosterone sulfate (DHEAS) were not available for analysis.

*Case report #2.*

This patient used opioids for 70 days before opioid tapering began. OME decreased by 70% between T0–T1, and opioids were fully tapered by T3. In parallel, estradiol levels were within the normal reference range during the whole study period (i.e., in contrast to Case Report #1 in which levels were extremely low at T0), initially getting lower between T0–T1 and then remaining unchanged at T3 compared to T1. Between T3 and six-month follow-up (i.e., T6), estradiol increased by 545%. For an overview of how endocrine measures changed over time during the study period, see **Figure S2**.

**Discussion S1**

Opioid administration may lead to decreased levels of estradiol [5]. Rhodin et al reported that women below 50 years of age treated with opioids had significantly lower levels of estradiol compared to controls [4]. The changes in levels of estradiol in our first case report may indicate that opioids had induced low levels of estradiol, which gradually was reversed as opioid tapering was initiated and oral morphine equivalents (OME) decreased over time. The largest increase in levels of estradiol coincided with the largest decrease in OME, i.e. during T1–T3 (**Figure S1**).

In contrast, for our second case report, changes in levels of estradiol after opioid tapering was started were not as clear. Interestingly, despite a reduction in OME between T0–T3, levels of estradiol decreased during the same period. However, by T6 levels of estradiol had increased, perhaps mirroring that by T3 opioids had been fully tapered (**Figure S2**).

The differences between the case reports may, in part, perhaps be explained by that we did not account for what point in their menstrual cycle case reports were, when blood samples were drawn. This is a weakness which may have impacted the levels of estradiol we report. However, these case reports may preliminarily illustrate how opioid tapering may affect levels of estradiol in premenopausal women.

Levels of follicle-stimulating hormone (FSH) and luteinizing hormone (LH) in both case reports increased after opioid tapering started, similarly to the results found for postmenopausal women in our main paper. This is in line with previous studies which have reported suppressed levels of LH due to opioid treatment [1; 2]. However, by the end of the study period at twelve-month follow-up (i.e., T12), only levels of LH remained elevated in the first case report, whereas FSH decreased to levels lower than that seen at tapering start.

Only one of our case reports had data on DHEAS and PRL available for analysis. Sustained-action opioids have previously been shown to lead to lower levels of DHEAS [3]. Seemingly congruent with this, levels of DHEAS in our case report increased quickly after opioid tapering started (**Figure S2**). Opioids are known to be able to induce hyperprolactinemia [1; 5]. Levels of PRL at T12 in our second case report were decreased compared to levels at T0 (**Figure S2**). This is congruent with the changes we found for levels of PRL in postmenopausal women in our main paper (changes which albeit were not significant). To summarize, our case report may offer preliminary insights into the changes that may occur in both DHEAS and PRL levels after opioid tapering is initiated.

All in all, these two case reports illustrate what may occur in endocrine measures after opioid tapering is initiated in premenopausal women. Future longitudinal studies on the subject would do well to account for menstrual cycle status when blood samples are drawn.

## References

[1] Abou-Kassem D, Kurita GP, Sjøgren P, Diasso PDK. Long-term opioid treatment and endocrine measures in patients with cancer-related pain: a systematic review. Scand J Pain 2022;22(3):421-435.

[2] Buss T, Leppert W. Opioid-Induced Endocrinopathy in Cancer Patients: An Underestimated Clinical Problem. Advances in Therapy 2014;31(2):153-167.

[3] Daniell HW. DHEAS deficiency during consumption of sustained-action prescribed opioids: evidence for opioid-induced inhibition of adrenal androgen production. J Pain 2006;7(12):901-907.

[4] Rhodin A, Stridsberg M, Gordh T. Opioid endocrinopathy: a clinical problem in patients with chronic pain and long-term oral opioid treatment. Clin J Pain 2010;26(5):374-380.

[5] Vuong C, Van Uum SH, O'Dell LE, Lutfy K, Friedman TC. The effects of opioids and opioid analogs on animal and human endocrine systems. Endocr Rev 2010;31(1):98-132.

**Figure S1.** Changes in endocrine measures and oral morphine equivalents (OME) in mg per day during the study period for the first case report patient. Dehydroepiandrosterone sulfate and prolactin were not available for analysis in this patient. Please note that each graph has a double y-axis with endocrine measure to the left and OME to the right.

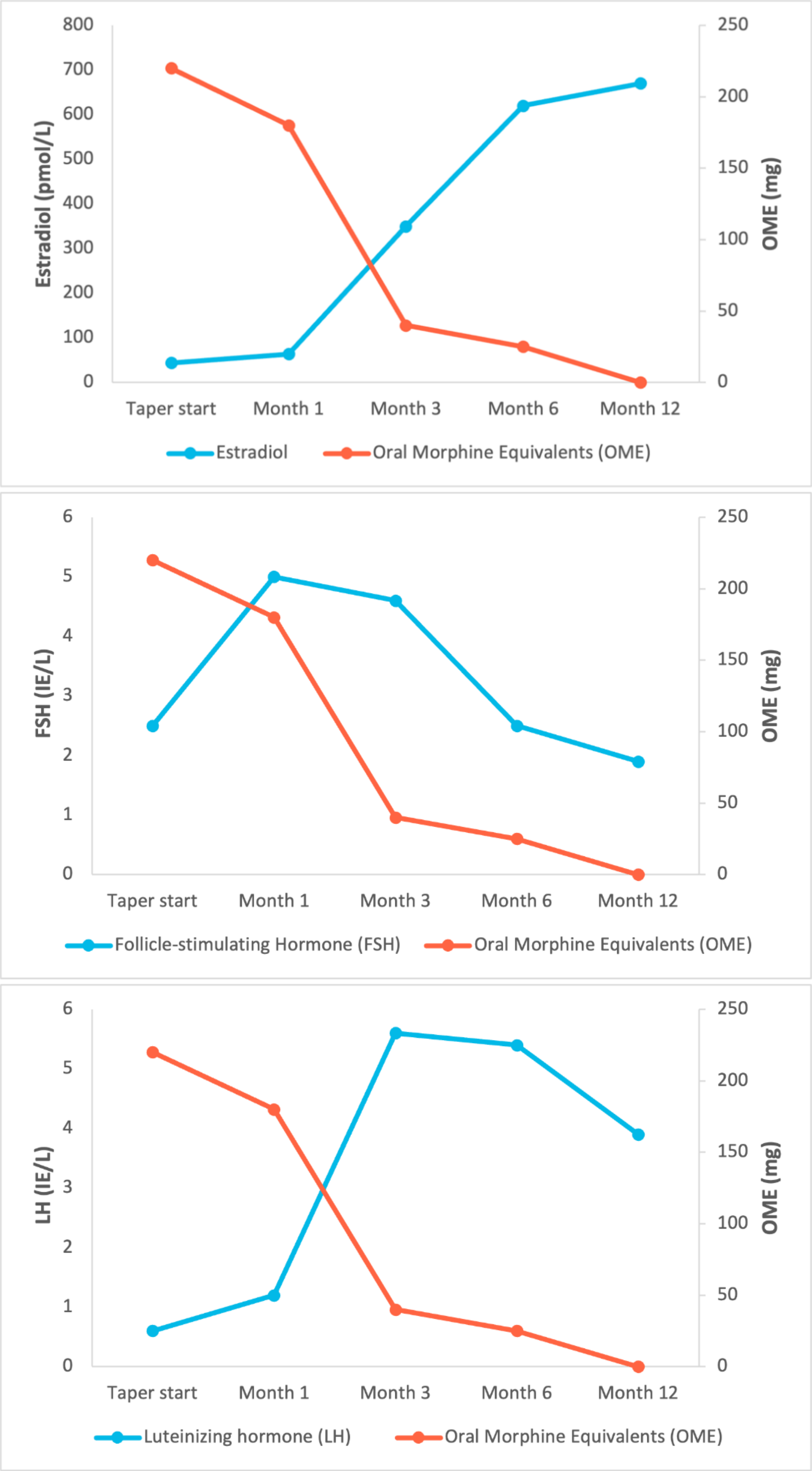

**Figure S2.** Changes in endocrine measures and oral morphine equivalents (OME) in mg per day during study period for the second case report patient. Please note that each graph has a double y-axis with endocrine test to the left and OME to the right.

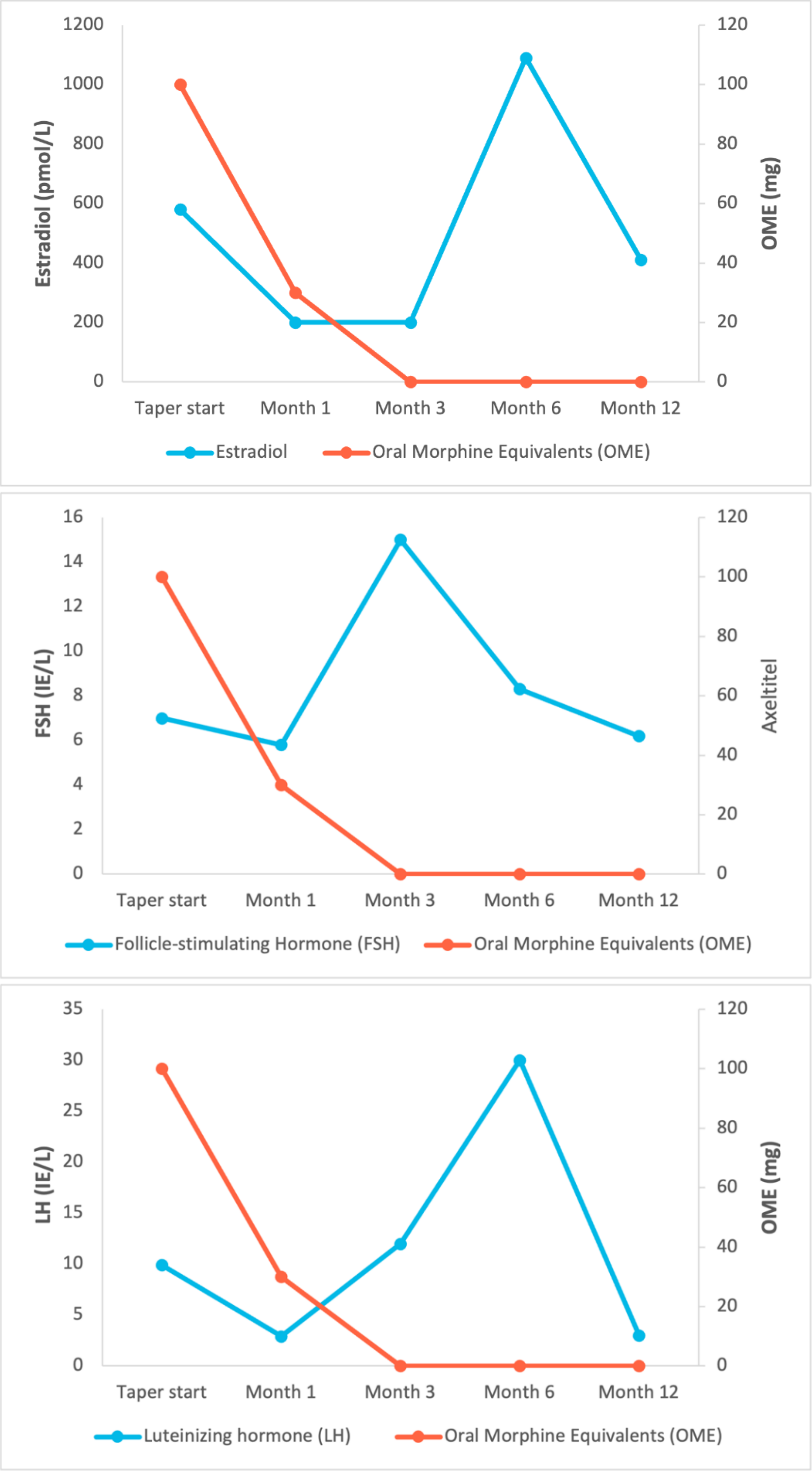

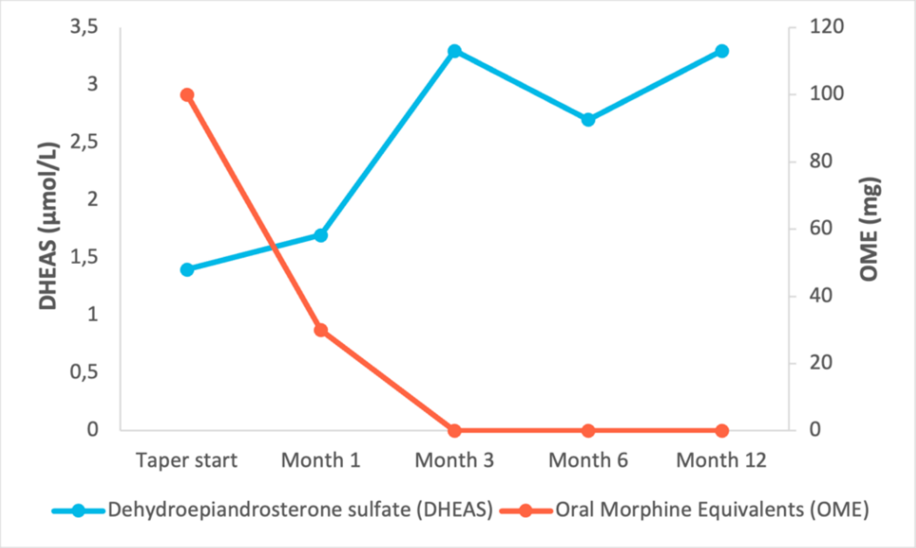
3,5
3
2,5
2
1,5
1
0,5
0
DHEAS (µmol/L)
120
100
80
60
40
20
0
OME (mg)
Taper start
Month 1
Month 3
Month 6
Month 12
Dehydroepiandrosterone sulfate (DHEAS)
Oral Morphine Equivalents (OME)

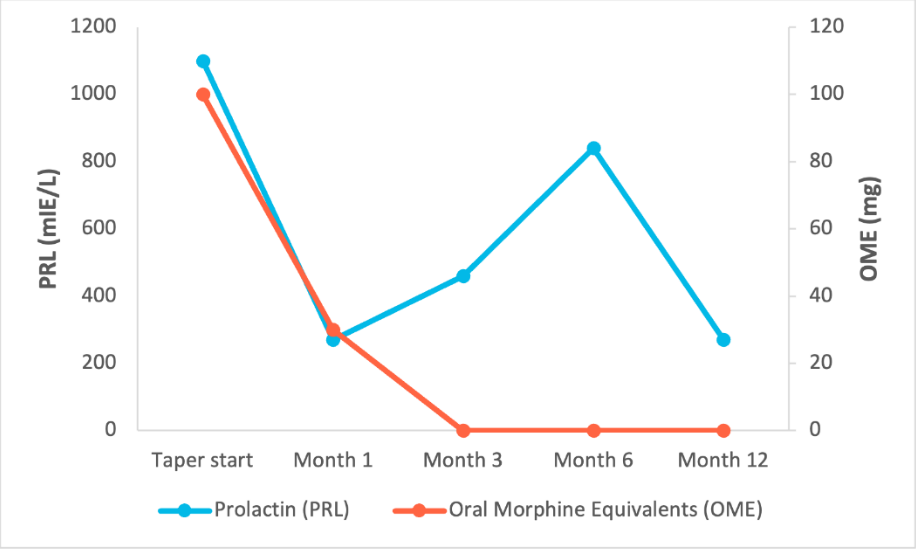
1200
1000
800
600
400
200
0
PRL (mIE/L)
120
100
80
60
40
20
0
OME (mg)
Taper start
Month 1
Month 3
Month 6
Month 12
Prolactin (PRL)
Oral Morphine Equivalents (OME)

## Tables

**Table S1.** Reference intervals for endocrine measures in men as specified by the clinical chemistry department at Linköping University Hospital according to sex and age. Blood samples were analyzed using immunoassays and electrochemiluminescence.

| Blood test | Years of age | Reference interval |
|---|---|---|
| **Testosterone, nmol/L** | <50 | 7.6–31 |
| | ≥50 | 4.6–31 |
| **Follicle-stimulating hormone (FSH), IU/L** | ≥18 | 1–13 |
| **Luteinizing hormone (LH), IU/L** | ≥18 | 1.7–8.6 |
| **Dehydroepiandrosterone sulfate (DHEAS), µmol/L** | 35–44 | 2.4–12 |
| | 45–54 | 1.2–9.0 |
| | 55–64 | 1.4–8.0 |
| | 65–74 | 0.91–6.8 |
| | ≥75 | 0.44–3.3 |
| **Prolactin (PRL), mIU/L** | ≥1 | 65–405 |

**Table S2.** Reference intervals for endocrine measures in women as specified by the clinical chemistry department at Linköping University Hospital according to sex, age, and menopause status. Blood samples were analyzed using immunoassays and electrochemiluminescence.

| Blood test | Menopause (Y/N) | Reference interval |
|---|---|---|
| **Estradiol, pmol/L** | Yes | <190 |
| | No | 90–1160 |
| **Follicle-stimulating hormone (FSH), IU/L** | Yes | 25–135 |
| | No | 1.7–22 |
| **Luteinizing hormone (LH), IU/L** | Yes | 7.7–59 |
| | No | 1.0–96 |
| | **Years of age** | **Reference interval** |
| **Dehydroepiandrosterone sulfate (DHEAS), µmol/L** | 35–44 | 1.6–9.2 |
| | 45–54 | 0.96–7.0 |
| | 55–64 | 0.51–5.6 |
| | 65–74 | 0.26–6.7 |
| | ≥75 | 0.33–4.2 |
| **Prolactin (PRL), mIU/L** | ≥1 | 65–465 |
